# Calculating Within-Pair Difference Scores in the Co-twin Control Design. Effects of Alternative Strategies

**DOI:** 10.1007/s10519-024-10196-9

**Published:** 2024-08-23

**Authors:** Juan J. Madrid-Valero, Brad. Verhulst, José A. López-López, Juan R. Ordoñana

**Affiliations:** 1 Department of Human Anatomy and Psychobiology, University of Murcia, Murcia, Spain; 2 Murcia Institute of Biomedical Research, IMIB-Arrixaca, Murcia, Spain; 3 Faculty of Psychology, University of Murcia, Campus de Espinardo, 30100 Murcia, Spain; 4 Department of Psychiatry and Behavioral Sciences, Texas A&M University, College Station, TX USA; 5 Department of Basic Psychology and Methodology, University of Murcia, Murcia, Spain

**Keywords:** Causality, Co-twin study, Statistics, Twin studies

## Abstract

Co-twin studies are an elegant and powerful design that allows controlling for the effect of confounding variables, including genetic and a range of environmental factors. There are several approaches to carry out this design. One of the methods commonly used, when contrasting continuous variables, is to calculate difference scores between members of a twin pair on two associated variables, in order to analyse the covariation of such differences. However, information regarding whether and how the different ways of estimating within-pair difference scores may impact the results is scant. This study aimed to compare the results obtained by different methods of data transformation when performing a co-twin study and test how the magnitude of the association changes using each of those approaches. Data was simulated using a direction of causation model and by fixing the effect size of causal path to low, medium, and high values. Within-pair difference scores were calculated as relative scores for diverse within-pair ordering conditions or absolute scores. Pearson’s correlations using relative difference scores vary across the established scenarios (how twins were ordered within pairs) and these discrepancies become larger as the within-twin correlation increases. Absolute difference scores tended to produce the lowest correlation in every condition. Our results show that both using absolute difference scores or ordering twins within pairs, may produce an artificial decrease in the magnitude of the studied association, obscuring the ability to detect patterns compatible with causation, which could lead to discrepancies across studies and erroneous conclusions.

**Supplementary Information:**

The online version contains supplementary material available at 10.1007/s10519-024-10196-9.

## Introduction

Twin registries are an invaluable resource (Odintsova et al. 2018) for disentangling the relative role of genetic and environmental factors in all kinds of phenotypes (Friedman et al. 2021; Knopik et al. 2017). Twin data are not only useful for classic heritability analyses but can also be used to test a variety of alternative hypotheses. Among other purposes, twin data can be especially useful to test for causal associations (Gonggrijp et al. 2023; McAdams et al. 2021; McGue et al. 2010). A causal relationship between an exposure and an outcome implies that the former (e.g., risk factor) causes the latter (e.g., a disease or disorder). However, an association between an exposure and an outcome might be non-causal when the relationship is due to a confounding factor that influences both (Kendler et al. 1993), and determining whether an association is truly causal is frequently an elusive task. There is a wide consensus that randomized experiments are the gold standard for testing for causality. However, quite often the hypothesized causal variable cannot be properly manipulated due to practical, methodological, or ethical constraints (Verhulst & Estabrook 2012).

Co-twin control studies have been proposed as an alternative quasi-experimental method to facilitate causal inference (Gonggrijp et al. 2023; McAdams et al. 2021; McGue et al. 2010; Verhulst and Estabrook 2012). Although they are not equivalent to a randomized experiment, they give us a powerful and elegant design which allows a suitable control for a wide range of confounders, both genetic and environmental, known and unknown (Kendler 2017; Vitaro et al. 2009). That is, twin studies cannot prove that an exposure causes an outcome, but they can show whether the former continues to predict the latter after accounting for the effect of shared aetiology (McAdams et al. 2021). The logic of the co-twin design is based on a close match between members of a twin pair, which includes, by definition, genetic and shared environmental factors. Therefore, twin pairs discordant for an exposure can be selected and the probability of experiencing the outcome compared. Alternatively, twins can be selected for outcome discordance in order to analyse the probability of having been exposed to the hypothesized risk factor (Goldberg and Fisher 2005).

A co-twin approach is commonly used to examine potentially causal associations observed at individual level (i.e., without regard to twin-pair membership). The co-twin approach may identify several alternative interpretations of the observed correlations. First, the correlation is not causal, but likely due to familial or shared environmental confounding (e.g., socioeconomic status). In this case, associations between the exposure and outcome variables observed at the individual level will be the same in dizygotic (DZ) or monozygotic (MZ) twin pairs. Second, the correlation is not causal, but likely due to genetic confounding. Such would be the case when the association between the exposure and outcome variables remains significant for DZ twins but disappears in MZ twins. Finally, the association remains significant at all levels, individual or within DZ/MZ pairs, which is consistent with a causal effect. Therefore, if we artificially underestimate the correlations, it will be more difficult to identify potentially causal associations. Scenarios 1 and 2 may depict different degrees of confounding, which can be total or partial depending on the within-DZ/MZ pair effect (Kendler et al. 1993; Kujala et al. 2002; McGue et al. 2010).

There are various approaches to operationalise discordance in a co-twin control study. One way is to construct a dichotomous (discordant/concordant) variable, but in doing so one is necessarily discarding information about the degree of discordance. Alternatively, it is possible to calculate a continuous difference score between the members of a twin pair. Continuous measures are calculated by subtracting the score of one twin from the score of the co-twin. The logic behind this approach can be summarized with the following formula: $E\left(\Delta {Y}_{i}\right)={\beta }_{w }E\left(\Delta {X}_{i}\right)$. The ${\beta }_{w}$ coefficient represents the expected difference between Twin 1 (T1) and Twin 2 (T2) on the outcome variable for each unit of change in the difference between T1 and T2 on the exposure variable (Vitaro et al. 2009). Correlations, different types of regression analyses, or structural equation modelling can then be used to analyse the association between difference scores on the exposure and the outcome to identify possible causal relationships (McAdams et al. 2021; Vitaro et al. 2009).

One of the most prominent differences is that for the dichotomous discordance strategy, a cut-off to select discordant twin pairs needs to be established. That is apparently straightforward for some conditions (e.g., T1 smokes but T2 does not), although reality tends to be not that simple (e.g., T1 started smoking last year and still does while T2 quit six months ago after 15 years of smoking). Using the difference scores approach allows researchers to include subtle gradient differences within twin pairs, thus preserving statistical power. Further, this retains the complex nuances inherent in continuously measured conditions such as neuroticism, weight, or sleep quality.

At first glance, it seems that the quantitative difference approach would be more appropriate when feasible. Yet, it also has some methodological boundaries that have not received enough attention to date. Specifically, difference scores are frequently computed in one of two ways: (1) as relative differences where the sign of the difference indicates which twin (T1 or T2) has the largest score on the phenotype; or (2) absolute differences where just the magnitude of the difference is considered. This differentiation may have major implications when analysing associations between variables that could be causally related.

Advice regarding how to compute within-pair difference scores in a co-twin design is scarce and contradictory. Rovine (1994) postulated that *“*when birth order is part of the hypothesis, a relative difference score (maintaining the birth order through the sign of the difference) is appropriate. When any difference is of importance and it does not matter which sibling has more of the characteristic, an absolute difference may be more appropriate*”.* Turkheimer and Waldron (2000) also stated that absolute differences can be computed when no ordering of the siblings is available or desirable. Other researchers, however, have suggested the use of relative differences computed by assigning twins randomly as T1 and T2 respectively (Vitaro et al. 2009; von Stumm and Plomin 2018). Nevertheless, both absolute and relative difference scores are common in the literature: (Barclay et al. 2013; Brendgen et al. 2017; Calais-Ferreira et al. 2021; Castelbaum et al. 2020; Isaksson et al. 2022; Kim et al. 2022; Klump et al. 2000; Korotana et al. 2018; Lundin Remnélius et al. 2022; Mosing et al. 2016; Torgersen and Janson 2002; von Stumm and Plomin 2018) Yet, the justification for using absolute or relative differences is often not clearly stated or even discussed in the manuscript. Furthermore, it is sometimes not clear if absolute difference scores are calculated for both the outcome and the exposure or just for one of them.

Relative difference scores appear to have an obvious advantage compared to absolute differences, given that they are not only informative about the magnitude of the difference (even though the mean tends to 0), but also about the direction of such difference. Yet, the magnitude of the association using relative difference scores with twins randomly assigned as T1 or T2 could differ from the use of absolute difference scores since the variability is reduced in the case of absolute as compared to relative difference scores. An alternative could be the ordering of twins such that the twin with the highest score is always assigned as T1. This should produce the same estimate of the regression slope as random assignment, for the line constrained through the origin (Carlin et al. 2005).

However, all those propositions remain speculative since no comparative exploration has yet been made about the discrepancies between the different approaches to data transformation. Results from co-twin studies using quantitative difference scores could vary depending on the type of scores (relative/absolute) and the twin-pair organisation (random/ordered) chosen. The aim of this study is to compare the results obtained by different methods of data transformation when performing a co-twin control study and to test how the association changes using each of those approaches. To that aim, we simulated different scenarios for a co-twin study, and we compared the association between two simulated variables in a co-twin design by varying the order of the twins for one of the variables (twins ordered such that T1 has the highest score in Variable 1, from 100 to 0% of the cases) and the kind of score (relative/absolute). This study aims to serve as a reference for performing a co-twin analysis by highlighting potential issues of selecting the either strategy, especially for those less familiar with the twin methodology.

## Method

A simulation study was performed using R version 4.0.4 (R Core Team 2021). The script for this simulation is available at (https://osf.io/ms4uw/?view_only=c4b49846af38401cbe788a4bc9100678). Data was created to simulate a scenario with two continuous variables (hereon V1 and V2) for pairs of twins (hereon T1 and T2). Data for 10,000 MZ twin pairs and 10,000 DZ twin pairs were simulated with fixed means (equal to 0 for both twins). These data were generated from a Direction of Causation (DoC) model (Duffy and Martin 1994; Heath et al. 1993; Verhulst and Estabrook 2012). We set the additive genetic factors (A) of V1 to 0.33, common-shared environmental factors (C) to 0.33 and non-shared environmental factors (E) to 0.34, whereas for V2 we set A to 0.50, C to 0, and E to 0.50 to maximize the statistical power of the DoC model. We simulated data for three different scenarios with different correlations: high causal path (r_V1,V2_ = 0.6), medium causal path (r_V1,V2_ = 0.3) and low causal path (r_V1,V2_ = 0.1). Additionally, we also simulated data with a high causal path and adding genetic confounders (genetic correlation [rA: 0.3]). The same simulations were performed for common-shared environmental confounders (shared environment correlation [rC: 0.3]) and non-shared environmental confounders (non-shared environment correlation [rE: 0.3]).

To test the main hypothesis of this study, difference scores (i.e., V1T1–V1T2 and V2T1–V2T2) were calculated. Pearson’s correlation between the intra-pair differences for both variables were then estimated both using absolute and relative difference scores. Difference scores were calculated for a range of scenarios by intentionally modifying the frequency of twin pairs where T1 had a higher score than T2 on V1, from 100% (T1 is always the highest score) to 0% (T1 is always the lowest score) in 5% intervals. Note that the 100% and 0% conditions are almost equivalent to absolute difference scores and that the 50% condition would be equivalent to perfect random assignment of twin order, which are the main proposed strategies for absolute and relative difference scores (Vitaro et al. 2009). Regression models using absolute/relative difference scores were also fitted. Additionally a regression model including within-pair and between-pair effects was also fitted (see McGue et al. (2010) for full description).

### Real Data Demonstration

Data on height and weight from the Murcia Twin Registry (MTR) in Spain, were used to demonstrate the importance of differences between the methods. Description about the MTR, recruitment procedures, ethics, and data collection is provided elsewhere (Ordoñana et al. 2019). Height in centimeters (mean: 162.4; SD = 10.2) and weight in kilograms (mean: 72.5; SD = 13.8) were selected as they are known to be correlated. These variables were collected using both objective methods (50% of the sample) and self-reported. Data from 654 complete same sex twin pairs were used for this test.

## Results

**Fig. 1:**
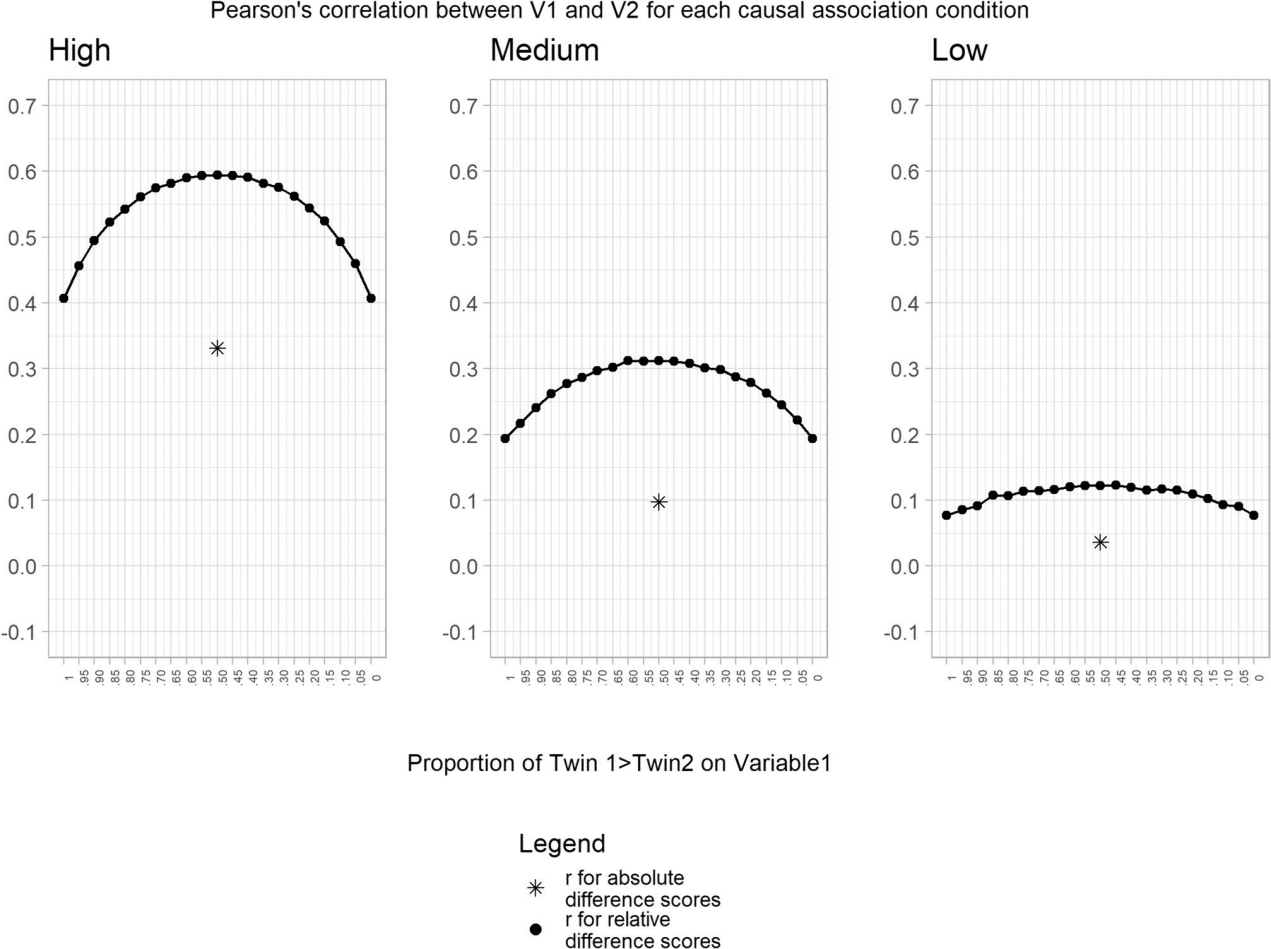
Association between variable 1 and variable 2 for each causal association condition. Note: The result for the absolute difference score is arbitrarily plotted at the 50% point

Figure 1 displays Pearson’s correlations across the different conditions (i.e., low, medium and high causal association between V1 and V2). The results for both MZ and DZ twin pairs are combined as there were no confounders (i.e., rA, rC and rE equal to 0) that would bias the estimated correlations. We found ample variation across the different scenarios of twin ordering (i.e., from 0 to 100%); variation that becomes more pronounced as the phenotypic correlation increases. For the high causal association condition Pearson’s correlation between difference scores ranged from 0.41 (0 and 100%) to 0.60 (50%); for the medium causal association condition ranged from 0.19 (0 and 100%) to 0.31 (50%); and for the low causal association condition the range varied between 0.08 (0% and 100%) and 0.12 (50%). The Pearson’s correlation between absolute difference scores for V1 and V2 always showed, again, a slightly lower value compared to the 0% and 100% conditions (0.33, 0.10 and 0.04 for the high, medium and low causal conditions respectively). In summary, the results show that: 1) correlations between relative difference scores are notably stronger than absolute difference scores; 2) the magnitude of the correlations using relative difference scores vary based on the proportion of time the value of twin 1 is greater than the value for twin 2; and 3) differences between relative and absolute difference scores are magnified as causal associations increase (Fig. 1).

**Fig. 2:**
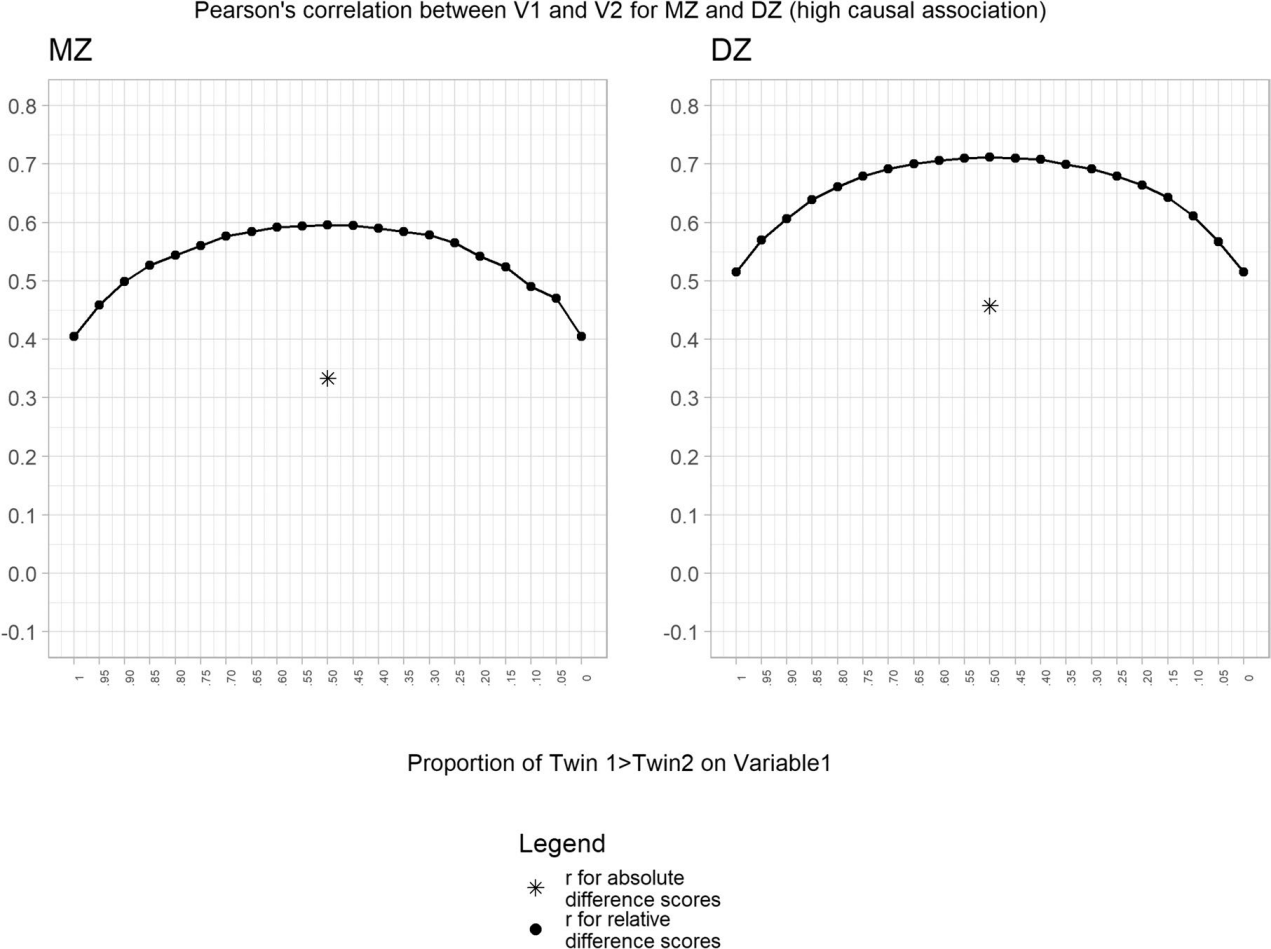
Association between variable 1 and variable 2 for the high causal association condition adding genetic confounders. Note: The result for the absolute difference score is arbitrarily plotted at the 50% point

**Fig. 3:**
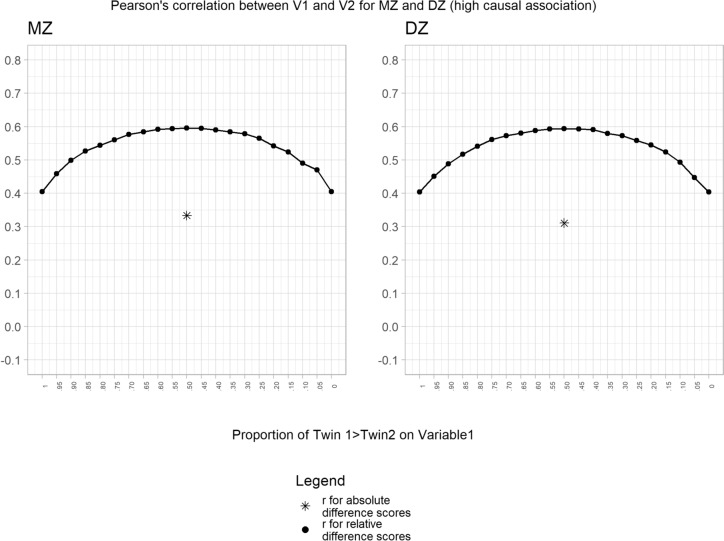
Association between variable 1 and variable 2 for the high causal association condition adding common-shared environmental confounders. Note: The result for the absolute difference score is arbitrarily plotted at the 50% point

**Fig. 4:**
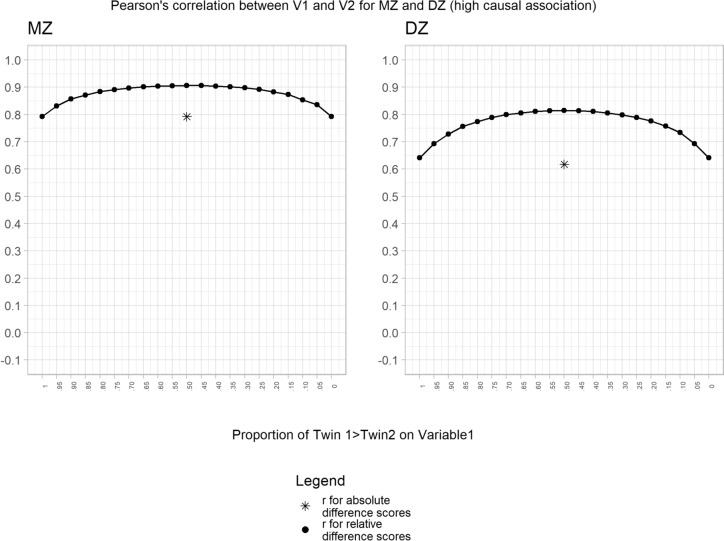
Association between variable 1 and variable 2 for the high causal association condition adding non-shared environmental confounders. Note: The result for the absolute difference score is arbitrarily plotted at the 50% point

We extended our analyses to examine the impact of genetic and environmental confounding. Using the high causal association condition (phenotypic correlation = 0.6) framework, we introduced a genetic correlation between V1 and V2 (rA = 0.3) to mimic confounding genetic factors. Our results again showed the same pattern of results for MZ and DZ twins, but notably the within-person DZ twin correlation was higher due to the fact that DZ twins share only 50% of their genetic factors (Fig. 2). The same pattern of results was found when we introduced common-shared environmental confounders (rC = 0.3), as expected, the within-person correlations were the same for MZ and DZ twins since common-shared environmental factors are fully controlled in both conditions (Fig. 3). Finally, when we introduced non-shared environmental confounders (rE = 0.3), we found higher correlations as compared to the simulated value (phenotypic correlation = 0.6) (Fig. 4). As expected this increase was higher for the MZ condition as previously reported in the literature (Frisell et al. 2012).

### Real Data Demonstration

Results using height and weight data from the MTR are very similar to those obtained using the medium causal association scenario. The matrix of correlations (MZ/DZ twins) was as follows:

$$\left(\begin{array}{ccccc}& Height-T1& Height-T2& Weight-T1& Weight-T2\\ Height-T1& 1& & & \\ Height-T2& 0.92/0.80& 1& & \\ Weight-T1& 0.55/0.57& 0.53/0.49& 1& \\ Weight-T2& 0.55/0.42& 0.57/0.54& 0.77/0.52& 1\end{array}\right)$$

Pearson’s correlation ranged from 0.20 (0%,100% and absolute difference scores conditions) to 0.29 (50% condition). These results show that depending on the method used (relative vs absolute difference scores) we can obtain a different value for the association between two phenotypes using the same empirical data (see Supplementary Fig. 1).

### Regression Models

Results for regression models comparing relative difference scores (only for 50% or 100% conditions) with absolute difference scores are presented in Table 1. For each of the simulated causal conditions as well as the real data analysis, the associations in the 50% relative difference condition were larger than in either the 100% relative difference condition or the absolute difference condition, thus providing more power to detect a causal effect. Another common analytical method for conducting cotwin analyses is to fit within-pair and between-pair effects (Carlin et al. 2005; McGue et al. 2010). Notably, the point estimates for the within-pair effects matched the 50% relative difference estimates. Similar results were found when different scenarios of confounding (genetic, shared-environmental, and non-shared environmental) were included, according to zygosity, within the high causal association condition (Suppl. Table 1). Note that all the regression models were fitted including the intercept term. However, regression models using difference scores must always be fitted without the intercept term as it has been previously reported (Carlin et al. 2005)—if the intercept term is included and twins are not randomly ordered then the results will be biased, underestimating the association, as shown in Table 1 and Supplementary Table 1.

**Table 1:** Regression models for the different causal scenarios (high, medium, and low causal association between variable 1 and variable 2) and real data from the Murcia twin registry

			Estimate	T value	P value	adjusted R^2^
	Relative differences 100%		0.185	63.03	< 0.001	0.17
High	Relative differences 50%		0.393	104.6	< 0.001	0.35
	Absolute differences		0.218	49.63	< 0.001	0.11
	Within between pair					0.45
		Within-pair	0.393	74.04	< 0.001	
		Between-pair	0.535	166.93	< 0.001	
	Relative differences 100%		0.095	27.93	< 0.001	0.04
Medium	Relative differences 50%		0.244	46.51	< 0.001	0.10
	Absolute differences		0.076	13.82	< 0.001	0.01
	Within between pair					0.14
		Within-pair	0.244	31.02	< 0.001	
		Between-pair	0.387	75.56	< 0.001	
	Relative differences 100%		0.040	10.94	< 0.001	0.006
Low	Relative differences 50%		0.099	17.44	< 0.001	0.01
	Absolute differences		0.030	5.13	< 0.001	0.001
	Within between pair					0.02
		Within-pair	0.099	11.38	< 0.001	
		Between-pair	0.167	28.51	< 0.001	
	Relative differences 100%		0.071	22.87	< 0.001	0.04
Real data	Relative differences 50%		0.142	7.16	< 0.001	0.08
(MTR)	Absolute differences		0.110	5.03	< 0.001	0.04
	Within between pair					0.33
		Within-pair	0.142	3.38	< 0.001	
		Between-pain	0.476	24.51	< 0.001	

Relative differences 100%: Highest score in variable 1 always assigned as T1; Relative differences 50%: Twins randomly assigned (i.e. T1 shows the highest score in variable 1 50% of the time)

All models were fitted including the intercept term

*MTR* Murcia twin registry

## Discussion

The results from our simulation study and data demonstration suggest that the method used to calculate difference scores has a large impact on the magnitude of the association in co-twin studies and thus any subsequent conclusions. When difference scores are calculated from continuous variables, correlations between relative difference scores are larger than correlations between absolute difference scores. Therefore, the appropriateness of the difference scores has major implications for the conclusions that are drawn from analyses of genetically informative designs such as the co-twin method (Kendler 2017; Kujala et al. 2002; McAdams et al. 2021; McGue et al. 2010). As such, the appropriateness of the transformations and procedures carried out in these kinds of studies is by no means a trivial issue, and results from co-twin studies may vary depending on the method used to calculate difference scores. In our real data demonstration, we showed that the magnitude of the association between two variables (i.e., height and weight) is considerably decreased (≈30%) depending on the analytical strategy (from 0.29 to 0.20). This has clear implications, since using the wrong strategy could lead to non-significant associations when in fact the relationship is present after controlling for familial factors. Additionally, this miscalculation could lead to erroneous conclusions regarding the presence of genetic and/or environmental confounders when comparing the estimates with those obtained from the analysis of non-related samples.

Although the scientific literature about the preference of absolute versus relative difference scores within co-twin studies is scarce, it has been proposed that relative difference scores would be appropriate if birth order is part of the hypothesis whereas absolute difference scores would be appropriate when it does not matter which twin has more of the characteristic (Rovine 1994). However, this last method could be counterintuitive since the direction of the association disappears, which could explain why other researchers have used or recommended relative difference scores on a regular basis (Vitaro et al. 2009; von Stumm and Plomin 2018). We have observed, using both simulated as well as real data, that the magnitude of the association between difference scores varies substantially depending on the strategy used to calculate those scores.

The main finding of our study is that when using absolute difference scores or relative differences with twins ordered within pairs, the correlation between difference scores tends to be artificially reduced, impeding the power to detect causal associations. This results from a drop in the variability of the difference scores produced by the restriction of the range of values when negative scores are transformed into positive ones. Such reduction in variability is known to lead to a decrease in the magnitude of the product-moment correlation (Cohen et al. 2003) and, hence the correlation estimates become lower as more negative values (or positive values depending on the sign of the association) for the within-pair differences are removed in one of the variables. In the same course of action, the use of absolute instead of relative difference scores (whichever the twin ordering) will lower correlation estimates as the loss of variability in this case occurs for both variables.

Behavioural research has shown the importance of taking into account small effects since psychological traits are likely influenced by a multitude of causes (Götz et al. 2021). For the co-twin control method, this implies that subtle variations yielded by the strategy chosen for data organisation/computation could alter the results substantially, leading to discrepancies among studies or erroneous conclusions. In the light of our results, the most appropriate estimate appears to be that obtained by ordering twins randomly and computing relative difference scores. By doing so, the direction of the association (as opposed to absolute values) is preserved, and the risk of a deflated correlation estimate reduced, at the same time that data manipulation is reduced to a minimum. This becomes increasingly important as within-twin cross-trait correlation increases since the difference between estimates escalates accordingly.

This strategy is also not free from possible biases. In particular, care should be taken to ensure that no association exists between the exposure/outcome studied and other variables that could be related to twin ordering. Of course, this would not be the method of choice if twin order forms part of the hypothesis. Furthermore, it is important to note that non-shared environmental factors could bias the results, as shown in our simulation including non-shared environmental confounders (Fig. 4), the association between variables increases and this increase is higher for the MZ condition. This has been previously addressed in several publications highlighting that this bias could be even higher as compared to the unpaired estimate (Frisell et al. 2012; Saunders et al. 2019). Accordingly, the theoretical motivation for utilising absolute difference scores should be carefully justified when reporting results from co-twin studies. Measurement error should also be considered in this context, whereas non-shared environmental confounders could increase the magnitude of the association, measurement error has the opposite effect and could lead to conclude that the causal association is confounded by familial factors (Frisell et al. 2012; Gustavson et al. 2024).

In summary, co-twin control designs can shed light on the causal associations between variables, but the selection of absolute or relative difference scores can have a major impact on the study’s conclusions. In this study, we examined the discrepancies across methods used to calculate within-pair difference scores both using simulated and real data. Our results show that when twins are not randomly assigned or using absolute difference scores might underestimate the association due to a reduction in variability, which could therefore mislead the conclusions of the study. We find that the use of relative difference scores, with twins randomly assigned, appears to be the method of choice in co-twin control designs, as long as twin birth order is not part of the hypothesis. Furthermore, given their potential relevance for the results, the procedure used for computing the difference scores should be reported clearly in the methods of co-twin control studies.

## Supplementary Information

Below is the link to the electronic supplementary material.

Supplementary file1 (TIF 4572 KB) Association between height and weight for MZ and DZ twins from the Murcia Twin Registry. The result for the absolute difference score is arbitrarily plotted at the 50% point.

Supplementary file2 (DOCX 20 KB)

## Data Availability

All the results can be replicated using the provided syntax, except results using real data from the MTR that is available under restrictions.

## Code Availability

Syntax for this study is available at (https://osf.io/ms4uw/?view_only=c4b49846af38401cbe788a4bc9100678).
