# Supplementary figures and images for: Calculating Within-Pair Difference Scores in the Co-twin Control Design. Effects of Alternative Strategies
Source: Behav Genet. 2024 Aug 23;54(5):426–35. doi: 10.1007/s10519-024-10196-9 (PMC11371853; doi:10.1007/s10519-024-10196-9)

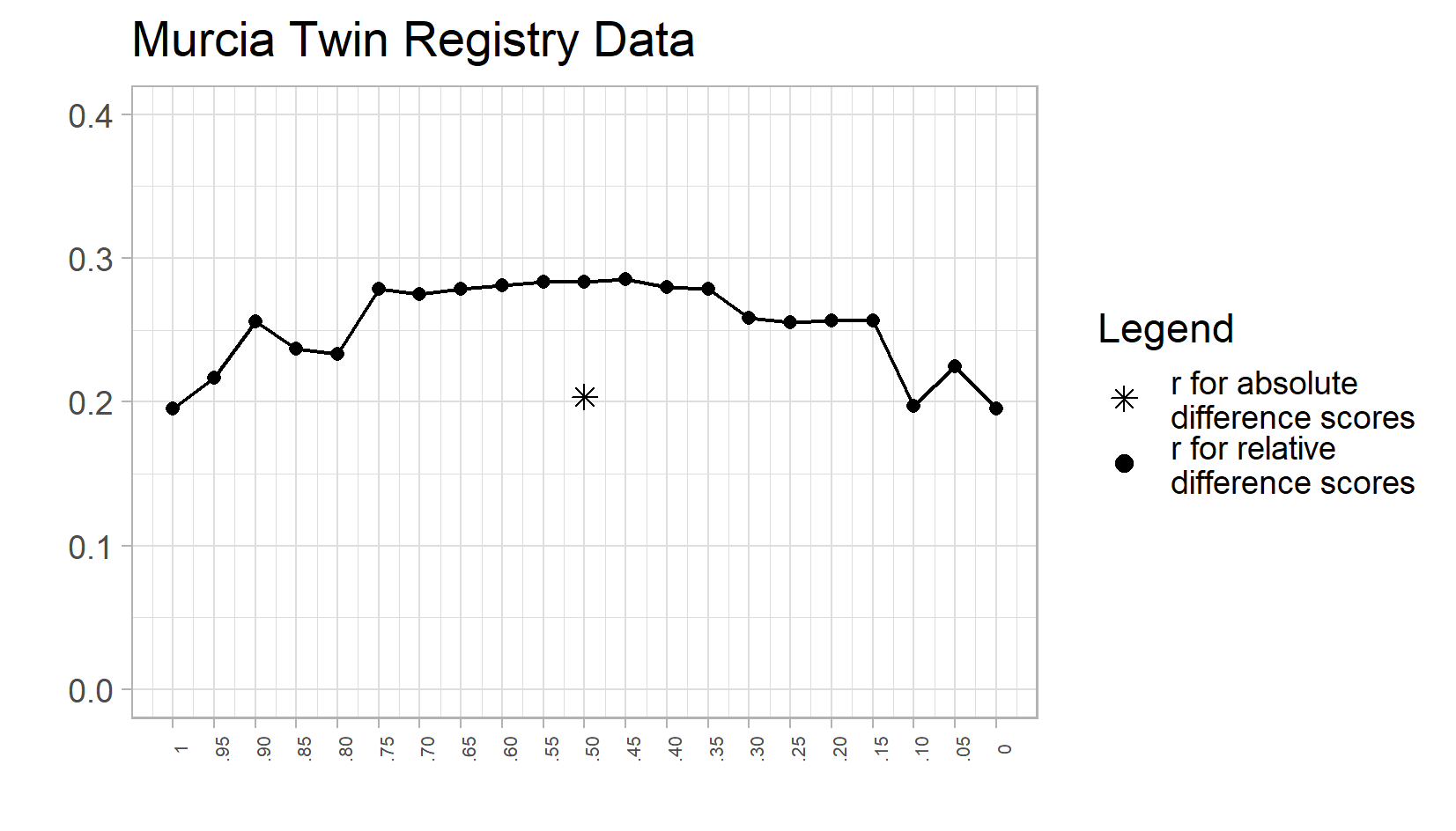

Supplement: Supplementary file 1 — Supplementary file1 (TIF 4572 KB) Association between height and weight for MZ and DZ twins from the Murcia Twin Registry. The result for the absolute difference score is arbitrarily plotted at the 50% point. [file 10519_2024_10196_MOESM1_ESM.tif]
