# Supplementary material for: Calculating Within-Pair Difference Scores in the Co-twin Control Design. Effects of Alternative Strategies
Source: Behav Genet. 2024 Aug 23;54(5):426–35. doi: 10.1007/s10519-024-10196-9 (PMC11371853; doi:10.1007/s10519-024-10196-9)
Supplement: Supplementary file 2 — Supplementary file2 (DOCX 20 KB) [file 10519_2024_10196_MOESM2_ESM.docx]

Table 1 (Suppl): Regression models for different scenarios of confounding (genetic, shared environment, and non-shared environment), according to zygosity, within a condition of high causal association between variable 1 and variable 2.

|  |  |  | **Estimate** | **T value** | **P value** | **adjusted R^2^** |
| --- | --- | --- | --- | --- | --- | --- |
|  | Relative differences 100% |  | 0.183 | 44.31 | <0.001 | 0.16 |
| **GENETIC** | Relative differences 50% |  | 0.395 | 74.21 | <0.001 | 0.36 |
| **CONFOUND** | Absolute differences |  | 0.220 | 35.35 | <0.001 | 0.11 |
| **(MZ)** | Within between pair |  |  |  |  | 0.62 |
|  |  | Within pair | 0.395 | 56.30 | <0.001 |  |
|  |  | Between pair | 0.533 | 171.2 | <0.001 |  |
|  | Relative differences 100% |  | 0.225 | 60.09 | <0.001 | 0.27 |
| **GENETIC** | Relative differences 50% |  | 0.423 | 101.30 | <0.001 | 0.51 |
| **CONFOUND** | Absolute differences |  | 0.272 | 51.43 | <0.001 | 0.21 |
| **(DZ)** | Within between pair |  |  |  |  | 0.62 |
|  |  | Within pair | 0.423 | 82.10 | <0.001 |  |
|  |  | Between pair | 0.548 | 161.20 | <0.001 |  |
|  | Relative differences 100% |  | 0.183 | 44.31 | <0.001 | 0.17 |
| **SHARED** | Relative differences 50% |  | 0.395 | 74.21 | <0.001 | 0.35 |
| **ENVIRONM.** | Absolute differences |  | 0.220 | 35.35 | <0.001 | 0.11 |
| **CONFOUND** | Within between pair |  |  |  |  | 0.45 |
| **(MZ)** |  | Within pair | 0.395 | 56.30 | <0.001 |  |
|  |  | Between pair | 0.533 | 171.20 | <0.001 |  |
|  | Relative differences 100% |  | 0.183 | 44.10 | <0.001 | 0.16 |
| **SHARED** | Relative differences 50% |  | 0.392 | 73.85 | <0.001 | 0.35 |
| **ENVIRONM.** | Absolute differences |  | 0.205 | 35.69 | <0.001 | 0.10 |
| **CONFOUND** | Within between pair |  |  |  |  | 0.62 |
| **(DZ)** |  | Within pair | 0.392 | 68.80 | <0.001 |  |
|  |  | Between pain | 0.549 | 168.50 | <0.001 |  |
|  | Relative differences 100% |  | 0.350 | 130.10 | <0.001 | 0.63 |
| **NON-SHARED** | Relative differences 50% |  | 0.461 | 214.20 | <0.001 | 0.82 |
| **ENVIRONM.** | Absolute differences |  | 0.401 | 129.89 | <0.001 | 0.63 |
| **CONFOUND** | Within between pair |  |  |  |  | 0.62 |
| **(MZ)** |  | Within pair | 0.461 | 214.20 | <0.001 |  |
|  |  | Between pain | 0.530 | 157.20 | <0.001 |  |
|  | Relative differences 100% |  | 0.468 | 76.42 | <0.001 | 0.41 |
| **NON-SHARED** | Relative differences 50% |  | 0.444 | 140.39 | <0.001 | 0.66 |
| **ENVIRONM.** | Absolute differences |  | 0.336 | 78.44 | <0.001 | 0.38 |
| **CONFOUND** | Within between pair |  |  |  |  | 0.62 |
| **(DZ)** |  | Within pair | 0.444 | 140.40 | <0.001 |  |
|  |  | Between pain | 0.548 | 153.80 | <0.001 |  |

Relative differences 100%: Highest score in variable 1 always assigned as T1; Relative differences 50%: Twins randomly assigned (i.e. T1 shows the highest score in variable 1 50% of the time).

NOTE: All models were fitted including the intercept term.
